# Impact of pediatric hypophosphatasia on behavioral health and quality of life

**DOI:** 10.1186/s13023-021-01722-7

**Published:** 2021-02-12

**Authors:** Elizabeth I. Pierpont, Jill H. Simmons, Katherine J. Spurlock, Ryan Shanley, Kyriakie M. Sarafoglou

**Affiliations:** 1 Department of Pediatrics, University of Minnesota Medical School, 2450 Riverside Avenue South, RPB 550, Minneapolis, MN 55454 USA; 2 Department of Pediatrics, Vanderbilt University Medical Center, Nashville, TN 37232 USA; 3 Biostatistics Core, Masonic Cancer Center, University of Minnesota, Minneapolis, MN 55455 USA; 4 Department of Experimental and Clinical Pharmacology, University of Minnesota College of Pharmacy, Minneapolis, MN 55455 USA

**Keywords:** Hypophosphatasia, Pediatric, Rare bone disease, Alkaline phosphatase, Behavior, Sleep disturbance, Attention deficit hyperactivity disorder, Quality of life

## Abstract

**Background:**

Hypophosphatasia (HPP) is a rare genetic disorder caused by loss-of-function mutations in the *ALPL* gene encoding tissue nonspecific alkaline phosphatase. It is characterized by defective bone mineralization associated with low alkaline phosphatase activity. Clinical features of pediatric HPP are highly variable, and can include premature loss of teeth, musculoskeletal problems, and impaired mobility. The effects of pediatric HPP on sleep, mood, regulation of attention and behavior, and other aspects of behavioral health have not been comprehensively studied.

**Methods:**

Parents of 30 children with HPP (14 females, 16 males) between the ages of 3 and 16 years (mean age = 8.0 years) enrolled in this cross-sectional survey-based study. Molecular genetic and biochemical testing as well as clinical records were reviewed to verify diagnosis of HPP. The cohort included 15 patients with a more clinically severe presentation of HPP who had received treatment with enzyme replacement therapy (asfotase alfa) and 15 children with less severe HPP who were treatment-naïve. Parents provided information regarding psychopathological comorbidity, emotional and behavioral well-being, and quality of life.

**Results:**

Clinically significant behavioral health challenges were evident in 67% of children with HPP. The most common behavioral findings included sleep disturbance and symptoms of attention deficit hyperactivity disorder (ADHD), each of which were observed ≥ 50% of individuals. Sleep disturbance, pain interference, poor behavioral regulation, and mood/anxiety symptoms were associated with reduced physical and psychosocial quality of life. Behavioral concerns were evident among children with HPP receiving asfotase alfa treatment as well as among children with clinically less severe disease who had not initiated therapy. Although most children in the cohort (77%) had age-typical development of adaptive skills, emotional and behavioral challenges were associated with weaker adaptive function.

**Conclusions:**

Children with HPP are at increased risk for ADHD symptoms and other behavioral health challenges. There is likely an under-recognition of these findings in clinical practice.

## Background

Hypophosphatasia (HPP) is a chronic, progressive bone mineralization disorder caused by loss-of-function variants in the *ALPL* gene encoding the tissue-nonspecific isoenzyme of alkaline phosphatase (TNSALP). TNSALP is required for the normal development of calcified tissues such as bones and teeth [1]. HPP is an extremely rare disease, with an estimated incidence of 1/300,000 for severe HPP phenotypes that manifest in the perinatal and infantile period and 1/6370 for the more frequent heterozygous forms [2]. Common clinical manifestations in children with HPP include premature loss of deciduous teeth, bone fractures, rickets, muscle weakness, and impaired mobility [3]. HPP manifests a remarkably wide range of severity, and is typically classified into subtypes based on patient age at presentation and diagnosis, and the presence or absence of skeletal disease [4]. Pediatric patients are typically categorized as having “perinatal” “infantile”, “childhood”, or “odonto” forms of HPP, although there is considerable clinical overlap associated with these categories. The perinatal and infantile subtypes are characterized by a higher mortality rate and profound skeletal hypomineralization in early life, whereas the later-onset subtypes are associated predominately with musculoskeletal or dental complaints. Traditionally, management of HPP focused on addressing the specific symptoms and complications of the condition. More recently, enzyme replacement therapy with a subcutaneous bone-targeted recombinant form of TNSALP (asfotase alfa) was approved for treatment of patients of all ages with a pediatric-onset HPP. Asfotase alfa has been established as a safe and effective therapy that improves survival, skeletal health, and respiratory function [5–7].

A spectrum of neurological and mental health symptoms has been associated with HPP. Seizures are a well-described feature among some patients with perinatal or infantile HPP, and are an indicator of disease severity and poor prognosis [8, 9]. The seizures are thought to arise due to altered neurotransmitter metabolism associated with TNSALP deficiency and central nervous system deficiency of pyridoxal 5′-phosphate (PLP) [10]. Although seizures are not typically present in HPP beyond perinatal/infantile onset, a recent retrospective study [11] provided evidence for a higher than expected prevalence of other neurological symptoms among individuals with HPP across the lifespan. Symptoms included fatigue, headache, sleep disturbance, neuropathic pain, and anxiety or mood disorders. These findings, in tandem with animal studies investigating TNSALP expression in the central nervous system [12–14], allude to an important role for TNSALP in brain development and function.

Studies investigating potential mechanisms involved in HPP brain pathophysiology offer evidence that TNSALP influences cellular processes important for myelin formation and synaptic plasticity [13, 15]. In humans, TNSALP activity occurs in cortical layers where integration from various brain regions occurs, suggesting a potential role in higher-order cognitive processes [16]. These findings suggest that altered TNSALP activity has the potential to disrupt circuitry underlying cognitive functions such as memory, attention, and regulation of emotions and behavior. Additionally, metabolic anomalies involving gamma-aminobutyric acid (GABA) and other neurotransmitters (dopamine and serotonin) may occur in HPP due to central nervous system deficiency of PLP (vitamin B6) [17]. In humans, dysregulation of these neurotransmitters has effects upon sleep, mood, and regulation of anxiety response [18–20]. Further, the clinical manifestations of HPP, such as reduced mobility, respiratory problems and weakness, may have notable effects on child development and emotional health. These chronic disease manifestations and their psychological effects are well-documented in various other pediatric conditions [21, 22]. Despite the physiological and disease burden vulnerabilities inherent in HPP, there is a lack of research prospectively investigating behavioral health among affected children.

To address the need for clarification regarding the impact of pediatric HPP on neurodevelopmental functioning and behavior, we collected detailed information from parents of children with HPP pertaining to their child’s behavioral health status. The study focused on quantifying psychopathological comorbidity, sleep habits, pain interference, social and emotional well-being, and adaptive function. We also assessed the relationship of these behavioral health domains to physical and psychosocial quality of life.

## Methods

### Participants

Parents/caregivers of children with HPP under the age of 18 years were invited to participate. Families were recruited through the University of Minnesota Masonic Children’s Hospital Hypophosphatasia clinic, emails distributed through the Coordination of Rare Diseases at Sanford (CoRDS) patient registry, study flyers distributed on social media or at clinic visits, or affiliation with patient advocacy groups (Soft Bones Foundation, MAGIC Foundation). Parents enrolling in the study provided written informed consent and completed a HIPAA authorization for release of medical records pertinent to the research. The study was approved by the University of Minnesota Institutional Review Board.

Parents of 30 eligible children (14 females, 16 males) provided information about their child with HPP. Children were at a mean age of 8.0 years (range 3.0–16.6 years) at the time of enrollment. The cohort included two sibling pairs. Medical records were requested from each patient’s medical providers to confirm diagnosis of HPP. Records confirming a pathogenic variant in the *ALPL* gene were available for 21 children in the study (Table 1). Of the remaining individuals, there were 3 children who had not undergone molecular testing, 5 children who had undergone molecular testing that confirmed an *ALPL* pathogenic variant but the results were unavailable for review, and one child whose molecular testing indicated an *ALPL* variant of uncertain significance not currently listed in genomic variant databases [23, 24]. For each of the individuals lacking documentation of a pathogenic *ALPL* variant, diagnosis of HPP was confirmed by biochemical test results indicating low alkaline phosphatase and/or elevated vitamin B6 levels *and* presence of at least one clinical feature of HPP (Table 1). Six participants were excluded from the study because their child had a diagnosis of another co-occurring genetic or neurological condition unrelated to HPP, or a history of very pre-term birth (prior to 32 weeks gestation). Four additional parents expressed interest but did not complete the surveys.

**Table 1:** Study cohort characteristics obtained through review of medical records and parent survey responses

Age (Years)	Sex	*ALPL* variant confirmed by molecular testing	Low alkaline phosphatase	Elevated vitamin B6	Clinical findings of HPP**	Asfotase alfa therapy	Duration of asfotase alfa therapy prior to enrollment	Mental health diagnoses***
3	F	c.1250A>G	X		PTL, SS			
3.5	M	Not tested	X	X	PTL, SS	X	2 years	
4	M	Not tested	X	X	PTL, C, MW, AG	X	2 years	OCD, sensory processing disorder
4.4	F	c.1259G>C	X	X	PTL			
4.6	M	c.892G>A		X				
4.9	F	c.1133A>T	X	X	PTL			
5.3	M	c.407G>A; c.984_986delCTT; *c.818C>T	X		PTL, SS	X	1 year	
5.5	F	Not available	X		PTL, C, MW	X	2 years (tapered off)	
5.6	F	c.1133A>T	X		PTL, LBMD, SS			Anxiety
5.6	F	c.1171delC; c.406C>T			PTL, SS, BLB, R, MW, AG	X	5.5 years	
5.8	F	c.1133A>T	X	X	PTL, BLB			
5.9	F	c.407G>A; *c.1213>C	X	X	PTL, SS, BLB, R, MW, AG	X	2 years	
5.9	M	c.1133A>T	X	X	PTL, LBMD			Anxiety
6	F	c.1471G>A; *c.1327G>T		X	PTL, C, CD, BLB	X	4 years	
6.2	M	c.1133A>T	X	X	PTL			
6.6	M	c.881A>C	X		SS, CD, MW			Autism spectrum disorder
7.7	M	c.1142A>G	X		PTL			Depression, disruptive behavior
7.7	M	c.976G>C	X		SS			Dyslexia
7.9	F	c.571G>A			PTL			
8	F	Not available	X		PTL, CD, BLB, MW, AG	X	2.6 years	
8.8	F	c.920C>T; c.1171C>T			PTL, LBMD, SS, CD, BLB, F, R, C, MW, AG	X	8.5 years	
9.5	M	c.346G>A; c.571G>A			PTL, LBMD, CD, R, MW, AG	X	2.6 years	
10.4	M	c.1250A>G	X	X	PTL, MW			
10.5	M	*c.1157G>A	X		F, MW, AG			ADHD, disruptive behavior
13.2	M	Not available	X		PTL, SS, CD, BLB, R, C, MW, AG	X	8 years	
13.3	M	c.340G>A	X		PTL, F, AG	X	Not available	ADHD, OCD, anxiety
13.6	M	Not available	X		PTL, BLB, F, R, MW, AG	X	0.8 year	
13.6	F	Not available	X	X	PTL, SS, CD, BLB, R, MW, AG	X	6 years	
15.4	M	Not tested	X	X	PTL, LBMD, R, MW, AG	X	1.6 years	Depression, dyslexia
16.6	F	c.526G>A; *c.371A>G	X	X	PTL, MW, AG			

*Variant of uncertain significance

**Clinical findings of HPP. *PTL* premature tooth loss, *LBMD* low bone mineral density/osteopenia, *SS* short stature, *CD* chest deformity, *BLB* bowing of the long bones, *R* ricket-like bone findings on x-ray, *F* recurrent or poorly healing fractures, *C* craniosynostosis, *MW* muscle weakness, *AG* abnormal gait

***Mental health diagnosis confirmed by medical documentation. *ADHD* attention deficit hyperactivity disorder, *OCD* obsessive–compulsive disorder

### Procedures

Parents electronically completed a set of questionnaires using University of Minnesota Research Electronic Data Capture (REDCap) survey tools [25] and the Pearson Assessment web-based test administration and scoring program. Medical records, which included laboratory and molecular testing, were reviewed for each child to confirm study inclusion criteria and clinical features.

### Measures

#### Demographics and medical history

Parents reported information regarding their child’s birth history, HPP diagnosis, symptoms, medications, developmental progression, previous mental health diagnoses, and educational placement.

#### Sleep

The Children’s Sleep Habits Questionnaire (CSHQ) is a 33-item questionnaire that measures clinical sleep complaints among school-aged children [26]. The CSHQ was originally developed for children ages 4–10 years, but application of this measure for assessment of preschool children and adolescents has also been validated in healthy and clinical populations [27–30]. Parents are asked to reflect on the past week to rate the frequency of problems in several domains, including concerns with sleep onset, sleep duration, resistance to going to bed, anxiety around sleep, night wakings, sleep-disordered breathing, parasomnias and daytime sleepiness. Raw scores across all items were combined to determine the total score, with higher scores reflecting more significant sleep problems. A cut-off score of ≥ 41 is typically recommended to classify a child as having significant sleep disturbance [26].

#### Pain

The 8-item Parent Proxy form of the PROMIS (Patient Reported Outcomes Measurement Information System) Pain Interference Scale was used to assess the impact of pain on a child’s daily functioning, behavior and mood [31]. Parents were asked to respond to statements about how pain influences their child’s behavior (e.g., “It was hard for my child to have fun when he/she had pain”) on a 5-point Likert scale ranging from “never” to “almost always.”

#### Emotional and behavioral functioning

The Behavior Assessment System for Children, Third Edition (BASC-3) Parent Report Form was used to assess behavioral and emotional functioning of children in home and community settings [32]. The BASC-3 provides age-adjusted standard scores along ten clinical scales (Hyperactivity, Aggression, Conduct Problems, Anxiety, Depression, Learning Problems, Somatization, Atypicality, Withdrawal, and Attention Problems) and four composite scales (Internalizing, Externalizing, Behavior Symptoms Index, Adaptive Functioning). Scores ≥ 60 are considered indicative of mild-to-moderate concerns within a domain; scores ≥ 70 typically indicate clinically meaningful impairment.

The ADHD Rating Scale-5, Home Version (ADHD-RS) was completed by parents of children ages 5–17 [33]. The ADHD-RS contains 18 items that directly correspond to the diagnostic criteria for ADHD in the Diagnostic and Statistical Manual of Mental Disorders, Fifth Edition (DSM-5). Symptoms were scored on a frequency scale ranging from 0 to 3. For any given child, a symptom was considered present if the item score was a 2 (occurring often) or 3 (occurring very often). Based on cut-offs recommended in DSM-5 (i.e., six of nine symptoms of inattention and/or six of nine symptoms of hyperactivity/impulsivity), the number of children meeting criteria for each of the three presentations of ADHD was determined.

#### Adaptive functioning

The Vineland Adaptive Behavior Scales, Third Edition (VABS-3) evaluates a child’s developmental milestones and behaviors enabling more independent functioning [34]. The VABS-3 measures a child’s abilities in three core domains: Communication, Daily Living Skills, and Socialization. Scores across all of these domains are combined to derive an overall Adaptive Behavior Composite score.

#### Quality of life

The Pediatric Quality of Life Inventory (PedsQL) Version 4.0 assesses health-related quality of life in terms of physical health functioning as well as psychosocial functioning across domains of emotional functioning, social functioning, and school functioning [35]. The parent-proxy form is suitable for children and adolescents 2–18 years old. This form requires parents to rate how frequently their child has had difficulties within the physical and psychosocial quality of life domains in the past month, with options ranging from “never” to “almost always”. Among children with major chronic conditions, a recommended cutoff score of 77 for children < 8 years, and 70 or children ≥ 8 years has been recommended to identify children with clinically meaningful impairment in quality of life [36].

### Statistical analysis

Statistical analysis was primarily descriptive. Where noted, Wilson confidence intervals were calculated for frequencies, and t-confidence intervals for mean differences. Spearman’s rank correlation coefficient (r_s_) was used to assess correlation between pairs of variables.

## Results

### Family, psychosocial, educational and medical history

Most children (n = 27) were living with biological parent(s), and 3 children had at least one adoptive parent. Twenty-three percent of families had at least one parent who completed high school/GED/Associate’s degrees, 23% had at least one parent who completed a Bachelor’s degree, and 53% had at least one parent who completed an advanced degree (e.g., Master’s, doctoral degree, law degree). Children attended a mixture of public schools (63%), private schools (10%), preschool/day care centers (10%), and homeschool or collaborative school (17%) settings. Eleven children (37%) received formalized educational supports or classroom accommodations (e.g., early intervention, speech therapy, occupational therapy, adapted physical education, special education instruction).

Consistent with the high degree of heterogeneity typically observed in HPP, children in this cohort presented with a wide range of clinical symptoms. Patients with a history of more severe musculoskeletal features had initiated enzyme replacement therapy more frequently than those with less severe symptoms (Table 2). Duration of asfotase alfa therapy ranged from < 1 year to > 8 years.

**Table 2:** Demographic and medical characteristics

Participant demographics	Untreated patients (n = 15)	Patients treated with asfotase alfa (n = 15)
	Mean (SD)	Mean (SD)
Age of child (Years)	7.2 (3.3)	8.7 (4.1)

	N (%)	N (%)
Gender (% Male)	8 (53%)	8 (53%)
Medical history		
Premature loss of deciduous teeth	10 (67%)	15 (100%)
Bowing of long bones in arms or legs	1 (7%)	8 (53%)
Recurrent bone fractures	1 (7%)	2 (13%)
Poorly healing bone fractures	0	1 (7%)
Short stature	4 (27%)	7 (47%)
Muscle problems (e.g., muscle weakness, gait problems)	4 (27%)	12 (80%)
Craniosynostosis	0	5 (33%)
Chiari malformation	0	3 (20%)
Seizures	0	2 (13%)
Chronic headaches	3 (20%)	8 (53%)
Fatigue	3 (20%)	11 (73%)

#### Psychopathological comorbidity

Previously established mental health diagnoses were reported by parents and confirmed by medical records in nine patients (30%), with five children having multiple diagnoses (Table 1). Mental health diagnoses were present among three children who had received asfotase alfa therapy and among six patients who had not received therapy. Anxiety was reported in three (10%), depression and/or disruptive behavior in two (7%), ADHD in two (7%), autism spectrum disorder in one (3%), and obsessive behaviors and/or sensory processing issues in two (7%) children. Learning disability (dyslexia) had been diagnosed in two individuals (7%).

### Behavioral health domains

#### Sleep

Ratings on the CSHQ indicated that more than half (17/30) of children with HPP (57%) were classified as “poor sleepers” when utilizing the CSHQ cut-off score of 41, as compared to 23% of healthy children in the normative sample [26]. CSHQ scores were similar for untreated children as compared to those receiving enzyme replacement therapy (mean difference: 1.9; 95% CI − 4.9 to 8.8). Total CSHQ scores were associated with patient age, such that older children and adolescents with HPP were more likely to have sleep difficulties than younger children (r_s_ = 0.37; *p* = 0.05). Examination of CSHQ subscale scores relative to patient age indicated greater concerns on the Daytime Sleepiness and Sleep Duration scales among the older children with HPP as compared to younger children.

#### Pain interference

Results from the 24 school-aged children in this cohort indicated that seven children (29%) experienced a higher than average level of pain interference during typical daily activities (T-score ≥ 60). Consistent with the greater prevalence of skeletal and muscle symptoms, patients with more severe HPP treated with asfotase alfa were reported to have greater pain interference than patients with less severe disease. Parent ratings of pain interference for these patients were on average 13 points higher than for untreated patients (mean difference: 13.0; 95% CI 2.5–23.5). Pain interference was also associated with age, with older children more likely to have pain interference than younger children (r_s_ = 0.39; *p* = 0.03).

#### Emotional and behavioral symptoms:

The average score of children with HPP was higher than the population mean (i.e., a T-score of 50) on all BASC-3 clinical scales, and it differed reliably from the normative sample mean on two of these scales. As a group, children with HPP showed greater symptomatology than healthy children on the Depression scale (M = 56.6, SD = 10.3; mean difference: 6.6, 95% CI 2.7–10.5) and the Somatization scale (M = 57.4, SD = 12.6; mean difference: 7.4, 95% CI 2.7–12.1), indicating more prevalent symptoms of irritability and depressed mood as well as physical complaints.

**Fig. 1:**
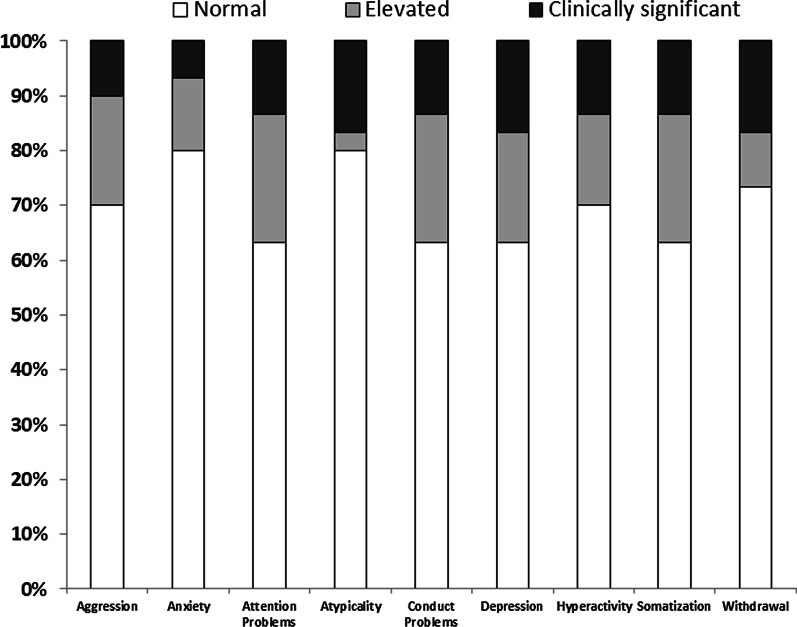
Classification of clinical severity of parent ratings of pediatric patients with HPP on the BASC-3 clinical scales. The shaded portion of each bar represents percentage of patients with elevated (light gray) and clinically significant (dark gray) scores on each scale. The white portion of each bar indicates percentage of patients scoring within normal limits

Examination of findings for individual patients revealed that BASC-3 scores for twelve patients (40%) were within normal limits on all of the clinical scales. Among the remaining patients, five patients (17%) had scores in the moderately elevated range (≥ 1 but < 2 SD) in at least one domain, and thirteen children (43%) had clinically significant scores (≥ 2 SD) in at least one domain (Fig. 1). A similar number of untreated patients (6/15) had clinically significant scores on at least one BASC-3 clinical scale as compared to patients undergoing enzyme replacement therapy (7/15).

#### ADHD symptoms

Results from the ADHD-RS indicated that 12 of the 24 school-aged children (50%; 95% CI 31–69%) met DSM-5 symptom criteria for ADHD based on their parent’s observation of their behaviors. This ADHD prevalence rate is notably higher than the 8.3% rate reported for the parent report version of this test within the ADHD-RS normative sample [33]. ADHD clinical symptoms were present among children with HPP who had not undergone treatment (45%) as well as those who had received treatment with asfotase alfa (54%). The inattentive presentation was the most common subtype of ADHD indicated by parent ratings (n = 7), followed by combined presentation (n = 3) and the hyperactive-impulsive presentation (n = 2).

Comparison of parent ratings on the ADHD-RS and the BASC-3 indicated broad agreement between these two measures. Ten of the 12 children whose ratings indicated ADHD presence on the ADHD-RS also had elevated scores (T-score ≥ 60) on the Inattention and/or Hyperactivity scales of the BASC-3. One child who had an elevated score on both scales scored just below the threshold for DSM-5 symptom criteria on the ADHD-RS. This finding suggests that, even when requiring stricter criteria for classifying presence of ADHD (i.e., abnormalities on *both* the ADHD-RS and the BASC-3), a substantial portion of the school-aged sample demonstrated significant concerns with attention and/or hyperactivity (42%).

#### Development of adaptive skills

**Fig. 2:**
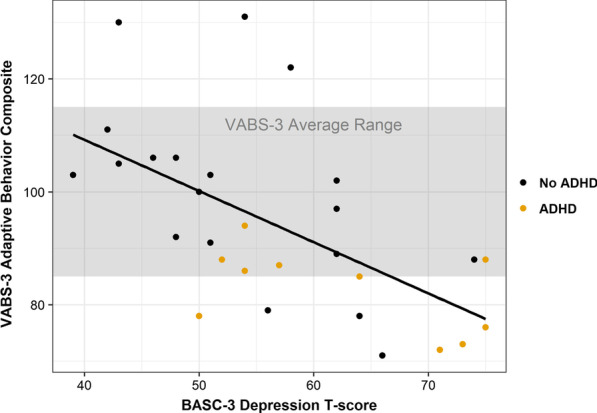
Symptoms of depression and ADHD were associated with weaker development of adaptive skills in pediatric patients with HPP. VABS-3 scores are standardized with an average score of 100 ± 15, with higher scores indicating better functioning. BASC-3 T-scores are standardized with an average score of 50 ± 10, with scores ≥ 60 indicating mild to moderate mood difficulties, and scores ≥ 70 indicating severe symptoms of depression. Children were classified as having clinical ADHD symptoms if ratings on the ADHD-RS met DSM-5 symptom criteria and they had an elevated scores (≥ 60) on the BASC-3 Inattention or Hyperactivity scales

Development of overall adaptive abilities was below average for 7 children (23%) and was within normal limits or above average for 23 children in the cohort (77%). The average VABS-3 score for children with HPP was only mildly lowered relative to the population mean score of 100. Scores were quite similar across subscales, which included communication (M = 94.6, SD = 14.6), daily living skills (M = 94.8, SD = 14.6), socialization (M = 95.7, SD = 16.2) and motor skills (M = 96.6, SD = 12.6). Most of the children who demonstrated delayed adaptive skills also demonstrated notable symptoms of inattention or hyperactivity. Children with lower VABS-3 scores were more likely to have symptoms of depression or ADHD (Fig. 2).

#### Quality of life

**Fig. 3:**
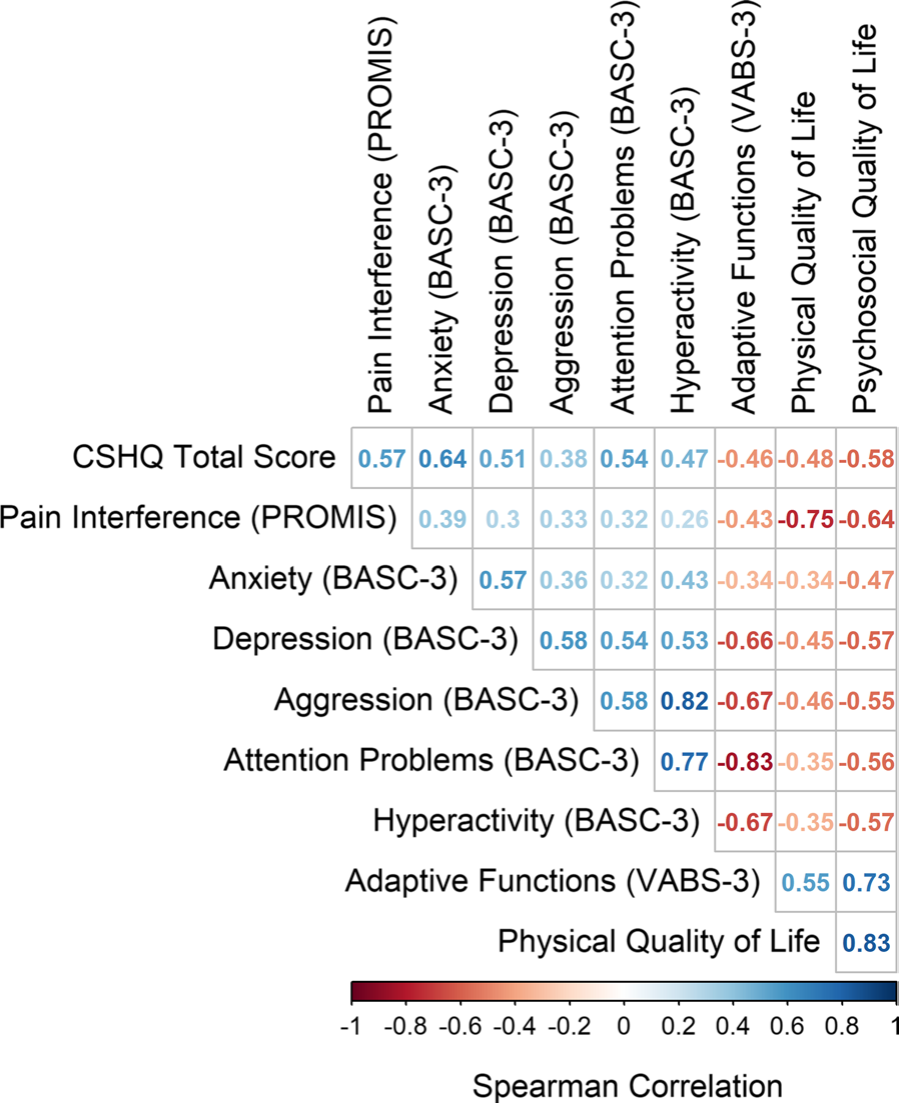
Correlations across domains in parent rating measures of behavioral health functioning of children with HPP. Problems with sleep, pain interference and behavioral health (mood, anxiety, attention problems/hyperactivity) showed moderate to high inverse correlations with adaptive functioning and physical and psychosocial quality of life among children with HPP. Positive correlations are depicted with blue and negative (inverse) correlations are depicted in red. Darker colors indicate stronger associations

Parent ratings of quality of life indicated clinically meaningful impairment in overall quality of life that were consistent with a major chronic health condition for 15 of the children with HPP (50%), including 6 patients who had not received enzyme replacement therapy for HPP. Interestingly, quality of life scores in the area of physical health functioning (Mean: 68.6, SD = 28.4) were similar to scores for psychosocial quality of life (Mean: 69.5; SD = 18.8) for children with HPP (mean difference = 0.9, 95% CI − 5.6 to 7.4). Parent ratings of both physical and psychosocial aspects of quality of life were inversely associated with children’s sleep problems and with level of pain interference in daily life (Fig. 3).

#### Behavioral wellness across domains

Examination of the scores of children with HPP across all of the domains of behavioral health (pain interference, sleep, emotional functioning, behavior, attention, adaptive skill development) indicated that 10 children (33%) with HPP in this study scored within normal limits on all of the measures administered. Elevated scores on the sleep questionnaire were the only significant behavioral concern for four additional children (13%), and none of these four children showed significant deficits in overall quality of life. For one child, symptoms of inattention were the only clinically significant behavioral problem, although reduced social and school-based quality of life were also observed in this individual. For the remaining 15 children (50%), challenges with behavioral health were relatively complex, spanning multiple domains. These behavioral health challenges were invariably associated with marked reduction in social, emotional, and/or school-based quality of life (i.e., PedsQL scores < 60 within at least one of these quality of life domains).

## Discussion

HPP is a rare metabolic disorder that is predominately associated with physical health manifestations including dental and musculoskeletal complaints. In addition to these common manifestations, the pathophysiology of HPP can also have neurological consequences. Altered production of TNSALP due to loss-of-function variants in the *ALPL* gene is associated with synaptic changes and altered production of neurotransmitters that may play an important role in stabilization of mood, sleep, and self-regulatory capacities [1]. Impaired transport of vitamin B6 across the blood–brain barrier may contribute to a presentation of seizure disorder among more severely affected individuals and potentially other wide-ranging consequences [9]. In the context of these potential central nervous system vulnerabilities, we conducted a cross-sectional investigation of behavioral health symptoms and quality of life in pediatric patients with HPP.

Analysis of emotional and behavioral health across a variety of domains indicated generally healthy, age-appropriate functioning among one in three children in this cohort. For another subset of children (13%), only mild concerns with sleep were evident, but these problems were not severe enough to cause major disruptions to quality of life. In contrast, among 50% of children with HPP studied, a more complex presentation of behavioral health concerns was observed. There was a high degree of overlap across behavioral health issues, with sleep and pain interference increasing with age and demonstrating moderate associations with mood and anxiety disturbance. In nearly all cases, these behavioral challenges were accompanied by reduced quality of life.

Despite the fact that children with HPP who had never received enzyme replacement therapy had fewer clinical findings of HPP and less pain interference than children given treatment, other behavioral health concerns such as poor sleep, attention problems and emotional difficulties (mood/anxiety disturbance) were observed at a similar rate in treatment-naïve children and those who had more significant clinical symptoms of HPP requiring therapy. This finding suggests that level of disease burden based on physical signs and symptoms may not fully account for the behavioral symptoms of HPP.

A notable finding of this study is that heightened risk for ADHD symptomatology appears to be associated with HPP. The percentage of school-aged children meeting DSM-5 criteria for ADHD based on parent ADHD-RS ratings in this cohort was 50%, which is more than 5 times the rate among children in the normative sample. Importantly, ADHD symptoms seem to be under-recognized by health care providers, as only two of the 12 children whose parent ratings reflected DSM-5 diagnostic criteria for ADHD had previously received a diagnosis of ADHD. A more focused study on ADHD symptomatology involving comprehensive clinical assessment and teacher rating scales is needed to reveal whether symptoms of inattention, hyperactivity and impulsivity are observed across settings or in the context of neurocognitive assessments.

Findings from our measure of adaptive function suggest that any adverse impact of pediatric HPP on the ability to perform daily life tasks independently is generally mild. Although direct cognitive assessment of all of the children in this study could not be obtained, scores on the VABS-3 adaptive measure (which is typically correlated with IQ) suggest that significant cognitive impairment would not typically be observed in children with HPP. Importantly, below average scores on the adaptive scale were associated with emotional and behavioral symptoms (i.e., depression and/or ADHD symptoms), indicating that a greater level of support for these symptoms may increase children’s ability to thrive and be more independent in their daily functioning.

## Limitations

The utilization of remote electronic survey administration methodology and the wide age range of the cohort (which included preschool children) limited the feasibility of obtaining children’s self-report ratings regarding their symptoms and quality of life. Obtaining perspectives directly from children and adolescents would enhance understanding of the emotional and functional impact of HPP. The electronic survey methodology and recruitment of participants through patient advocacy groups may also have led to greater inclusion of families with high educational attainment, which can impact generalizability of results. Measurement of disease severity is challenging in pediatric HPP as there is no validated scale of disease burden for this population. Disease-specific rating scales with regard to symptom severity and quality of life are greatly needed [37]. Further, studies employing more targeted evaluation of symptomatology (e.g., comprehensive clinical assessments of anxiety, ADHD or executive function) would constitute an important follow up to our study, which utilized primarily broad-based metrics.

Another limitation of this study is the fact that disease severity and treatment effects were not able to be separately assessed in this cohort. Enzyme replacement therapy has established benefits for improving clinical outcomes among patients with severe, debilitating HPP, and as such it is not possible to ethically study the natural history of behavioral health concerns among these patients in the absence of therapy. Behavioral assessment prior to the onset of treatment was not available for any of the children in this study, limiting any conclusions regarding the effect of therapy on these symptoms.

This study also provided only a cross-sectional view of this cohort. Longitudinal studies are necessary to determine whether behavioral health symptoms change over time as children with HPP develop, or if symptoms are modified upon initiation and/or maintenance of therapy.

## Conclusions

Standard HPP surveillance and treatment protocols for pediatric patients should involve screening and follow-up intervention for concerns related to neurodevelopmental and behavioral functioning. Clinicians should be aware that problems related to sleep duration and quality, ADHD symptoms and mood/anxiety disturbance may be more common than expected among affected individuals.

## Data Availability

The data that support the findings of this study are available from the authors upon reasonable request.
